# Complex Coronary Artery Lesions in a Young Woman With an Acute Myocardial Infarction and Genetically Confirmed Familial Hypercholesterolemia: A Case Report and Literature Review From a Developing Country

**DOI:** 10.7759/cureus.68212

**Published:** 2024-08-30

**Authors:** Kha M Nguyen, Sy V Hoang, Tai N Nguyen, Sang Q Ly, Vi T Dang, Trung I Ly, Hai P N Tran

**Affiliations:** 1 Department of Internal Medicine, Faculty of Medicine, University of Medicine and Pharmacy at Ho Chi Minh City, Ho Chi Minh, VNM; 2 Department of Internal Medicine, Faculty of Medicine, University of Medicine and Pharmacy at Ho Chi Minh City, Ho Chi Minh City, VNM; 3 Department of Interventional Cardiology, Cho Ray Hospital, Ho Chi Minh City, VNM

**Keywords:** ldl-r, gene, ldl-c, familial hypercholesteremia, myocardial infarction

## Abstract

Familial hypercholesterolemia (FH) is a genetic disorder characterized by elevated levels of low-density lipoprotein cholesterol (LDL-C) in the blood from an incredibly early age. This condition leads to the early development of atherosclerotic arterial diseases, which can manifest even in the first few decades of life. Mutations in genes related to the LDL receptor (LDL-R), apolipoprotein B (APOB), and proprotein convertase subtilisin/kexin type 9 (PCSK9) are the main molecular mechanisms causing familial hypercholesterolemia. This case involves a 44-year-old Vietnamese female who presented at the emergency department with chest pain and was diagnosed with acute myocardial infarction (AMI) complicated by cardiogenic shock. Clinical signs and an elevated LDL-C level pointed to prolonged exposure to high cholesterol. A Dutch Lipid Clinic Network (DLCN) score of 10 further supported the diagnosis of FH. The reverse T-stenting and small protrusion (TAP) technique was selected and successfully employed to stent the LMCA, left anterior descending artery (LAD) and left circumflex artery (LCx). This technique was chosen due to its simplicity and rapid execution, making it particularly suitable in situations of cardiogenic shock where time-consuming procedures should be avoided. Genetic testing confirmed a heterozygous pathogenic mutation in the LDL-R gene, corroborating the clinical diagnosis of FH. The patient's condition has gradually stabilized, and they have been discharged from the hospital. The patient is currently being monitored as an outpatient at the cardiology clinic. This case emphasizes the importance of considering FH in patients with premature cardiovascular events by applying the clinical diagnostic criteria and confirming by genetic analysis. It also highlights advanced interventional techniques for managing complex coronary lesions, such as reverse TAP.

## Introduction

Familial hypercholesterolemia (FH) is an inherited disorder characterized by high levels of low-density lipoprotein cholesterol (LDL-C), significantly increasing the risk of cardiovascular diseases at a young age [1-3]. It is an autosomal codominant genetic disorder with a prevalence that varies widely, but studies indicate that it is underdiagnosed globally. Familial hypercholesterolemia is caused primarily by mutations in the gene encoding the LDL receptor (LDL-R), with less frequent mutations in the apolipoprotein B (APOB) and proprotein convertase subtilisin/kexin type 9 (PCSK9) genes [4]. The prevalence of physical signs is relatively lower in contemporary heterozygous FH patients, therefore requiring reliance on serum LDL-C levels [5]. In developing countries in Southeast Asia, such as Thailand and Malaysia, the diagnosis of FH in patients with premature coronary artery disease (CAD) primarily relies on the clinical Dutch Lipid Clinic Network (DLCN) score. Genetic mutation testing has not yet been routinely implemented, possibly due to limited healthcare resources [6,7]. Genetic analysis is the gold standard for definitive diagnosis, providing the basis for appropriate management strategies and family screening [8,9]. 

Patients with FH are at a 20-fold increased risk of developing early CAD compared with the general population [2]. However, the condition is often not recognized until an acute event such as myocardial infarction or stroke occurs. Patients with acute myocardial infarction (AMI) who have FH often experience more severe coronary artery damage than those without FH due to prolonged high levels of LDL-C and early atherosclerosis [10]. This patient needs to be given an aggressive treatment strategy and close monitoring to reduce the risk of subsequent cardiovascular events. Coronary artery lesions due to atherosclerosis in patients with FH are typically more severe in terms of the number of affected coronary arteries as well as the degree of coronary artery calcification. Managing these patients is challenging not only in terms of controlling LDL-C levels but also in optimizing interventional procedures for their complex coronary artery lesions [3,11]. Prolonged exposure to elevated cholesterol levels in patients with FH often results in severe and diffuse coronary artery disease if not detected and treated early. In cases where patients develop cardiogenic shock due to complex coronary bifurcation lesions, particularly involving the left main coronary artery (LMCA), the lesions tend to be more complicated, often necessitating the use of two-stent techniques rather than one due to the high risk of side branch loss from the heavy atherosclerotic burden [12].

This case report presents a young female with early AMI related to FH with diffuse severity coronary artery treated via the reverse T-stenting and small protrusion (TAP) technique, a specialized method for complex coronary bifurcation lesions [13]. Through this, the imperative of screening for FH in young patients with AMI was emphasized. Furthermore, it introduces a technique that should be considered for treating severe diffuse CAD.

## Case presentation

A 44-year-old Vietnamese female presented to the hospital with acute central chest pain that started abruptly eight hours before admission. She described the pain as severe, pressing, and associated with sweating and shortness of breath. The patient had no significant medical history and did not smoke. There was no known family history of cardiovascular diseases or hyperlipidemia.

**Figure 1:**
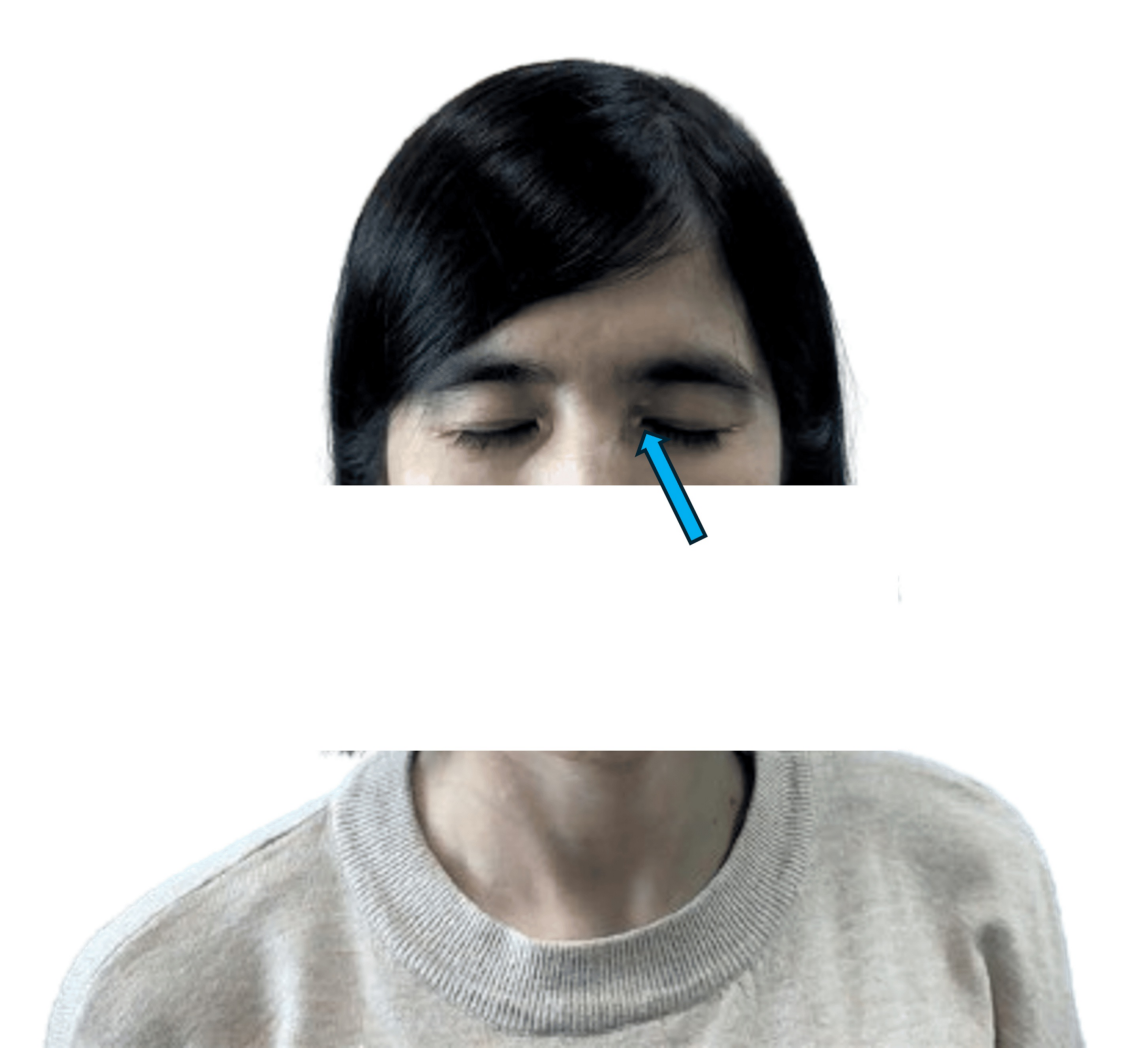
The xanthelasma sign on both eyelids The green arrow indicates the xanthelasma on the eyelid.

On admission, the patient was alert but in distress due to severe chest pain. The vital signs were as follows: heart rate of 110 beats per minute; blood pressure of 90/50 mmHg (supported by noradrenaline at 5 µg/min); respiratory rate of 26 breaths per minute; temperature of 37°C; and oxygen saturation (SpO_2_) of 95% on room air. Physical examination revealed clear heart sounds, moist rales in the lower half of both lung fields, and xanthelasma on both eyelids (Figure 1), indicating lipid metabolism disorders. The initial laboratory test results in Table 1 noted an extremely high serum LDL-C of 370 mg/dL.

**Table 1:** Initial laboratory tests at admission

Laboratory tests	Result	Normal range
Hemoglobin	145	120 - 170 g/L
White blood cells	15	4 - 11 G/L
Platelets	348	200 - 400 G/L
Blood urea nitrogen	19	7 - 20 mg/dL
Serum creatinine	0.9	0.7 - 1.5 mg/dL
Estimated glomerular filtration rate (eGFR)	68	> 90 mL/min/1.73 m²
Total cholesterol	451	140 - 239 mg/dL
High-density lipoprotein cholesterol	25	> 45 mg/dL
Low-density lipoprotein cholesterol	371	90 - 150 mg/dL
Triglycerides	143	35 - 160 mg/dL
Glycated hemoglobin (HbA1c)	5.6	4 - 7%
Free thyroxine	2.1	1.5 - 4.2 pg/mL
Thyroid-stimulating hormone	1.34	0.4 - 5 mIU/mL
High-sensitivity troponin I	11318	< 34 pg/mL

**Figure 2:**
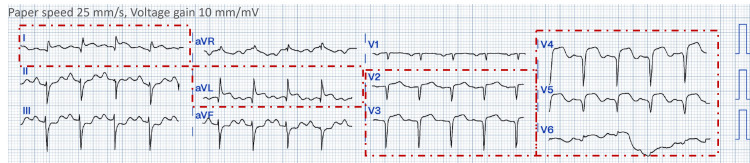
The patient's electrocardiogram at admission revealed sinus tachycardia; ST elevation from V2 to V6; DI; and aVL. The faint red dashed border indicates the leads with ST elevation.

The 12-lead electrocardiogram revealed diffuse ST-segment elevation in leads V1-V6, DI, and aVL (Figure 2). Transthoracic echocardiography revealed global hypokinesia of the left ventricle with an ejection fraction of 20% (Simpson's method).

The patient was diagnosed with extensive anterior ST-elevation myocardial infarction (STEMI) (Killip IV) and cardiogenic shock. She was immediately transferred to the catheterization laboratory for emergency coronary angiography, which revealed severe diffuse three-vessel coronary artery disease: subtotal occlusion of the LMCA, 90% stenosis in the proximal and distal LAD artery, 90% stenosis in the proximal LCx with total occlusion in the mid-segment, and 80% stenosis in the mid-right coronary artery (RCA) with subtotal occlusion of the posterior left ventricular branch (PLV). The Gensini score was calculated at 291.5 points (Videos 1, 2).

**Video 1:** The patient’s coronary angiography at the cath lab revealed right coronary artery stenosis.

**Video 2:** The patient’s coronary angiography at the cath lab revealed the anterior descending artery and left circumflex stenosis.

Given the cardiogenic shock and high-risk percutaneous coronary intervention (PCI), we provided additional support to the patient with an intra-aortic balloon pump prior to the procedure. The culprit lesion involved the LMCA with a complex true bifurcation classified as Medina (1,1,1). Consequently, we opted for a two-stent strategy from the outset.

The reverse TAP technique was employed for PCI to address the complex coronary anatomy. This technique involves (1) positioning a drug-eluting stent (DES) from LAD II into the LMCA via a small protrusion to ensure optimal coverage and minimal overlap; (2) deploying a second DES in the LMCA extending into the LCx; and (3) final kissing balloon inflation to optimize stent apposition and expansion (Video 3). This technique achieved TIMI grade 3 flow post-intervention. The patient was then transferred to the cardiac intensive care unit for further monitoring and management.

**Video 3:** Final result with the caudal view (RAO 15 CAU 30)

**Figure 3:**
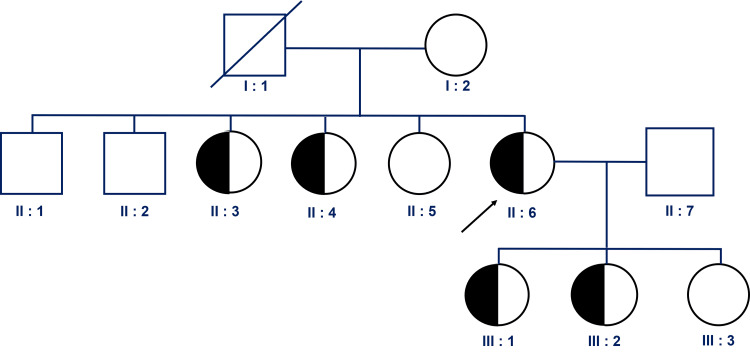
Pedigree and mutation analysis of the FH family Nine direct relatives of the patient (II6) were advised to undergo genetic mutation testing using the Sanger method. Among them, the patient’s two sisters (II3 and II4) and two daughters (III1 and III2) were found to carry the genetic mutation at position (LDL-R) .664T>C (p.Cys222Arg).

The patient showed significant improvement and was discharged after one week of hospitalization. Follow-up visits were scheduled to monitor her condition continuously. Genetic testing via next-generation sequencing (NGS) via the Illumina machine system (Illumina, Inc., San Diego, CA) identified a heterozygous pathogenic variant in the LDL-R gene NM_000527.5(LDL-R) .664T>C (p.Cys222Arg), confirming the diagnosis of FH. She continued to receive treatment according to national guidelines for AMI following coronary intervention. The patient’s first-degree relatives were also advised to undergo genetic screening for FH-related mutations to enable early detection and prevention of this genetic disorder. Figure 3 revealed the pedigree map with a pathologic mutation (LDL-R) .664T>C (p.Cys222Arg).

**Figure 4:**
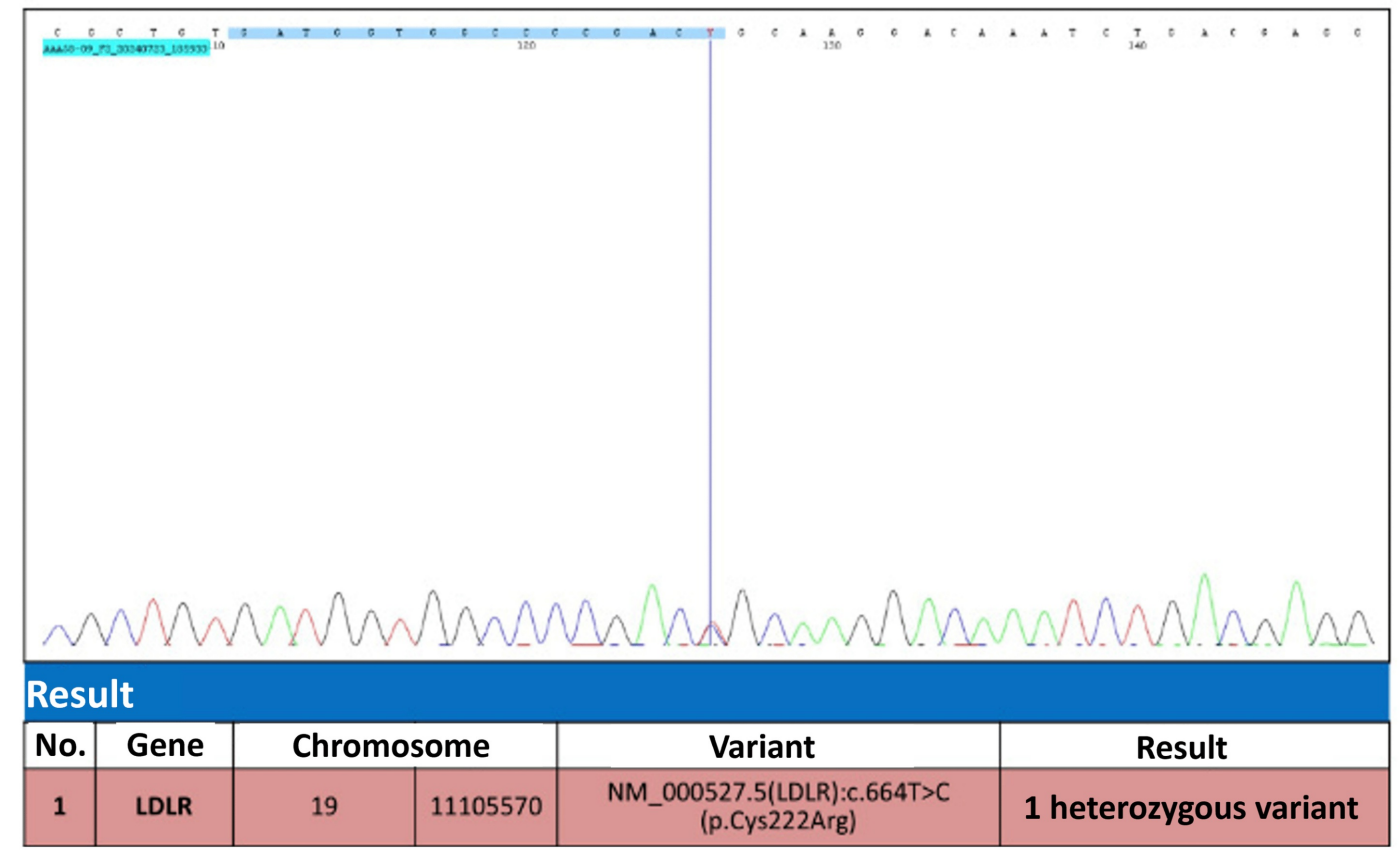
Sequencing results of chromosome 19 with a variant (LDL-R): c.664T>C The Sanger sequencing results for a direct relative of the patient (designated as II3 in Figure 3) revealed a heterozygous point mutation at nucleotide position 664, where T is replaced by C, resulting in the substitution of the amino acid cysteine with arginine at codon 222 of chromosome 19.

The results of the Sanger sequencing performed on a relative are shown in Figure 4.

## Discussion

This case illustrates the importance of considering FH in young patients who present with premature myocardial infarction. Familial hypercholesterolemia is an autosomal dominant disorder caused by mutations in genes involved in lipid metabolism, most commonly the LDL-R gene [4]. It is characterized by elevated LDL-C levels, leading to early atherosclerosis, and increased cardiovascular risk. The diagnostic criteria for FH are based on clinical factors, family history, and biochemical test results. The DLCN score is a valuable diagnostic tool, with a score ≥ 8 points confirming the diagnosis of FH [14].

In this patient, the diagnosis of FH was supported by her markedly elevated LDL-C levels and the presence of xanthelasma. The early onset of myocardial infarction and extensive CAD observed via angiography are typical complications of untreated or poorly managed FH [15]. The patient in this report scored 10 DLCN points, including two points for early myocardial infarction and eight points for very high LDL-C levels. Genetic testing confirmed a heterozygous pathogenic variant in the LDL-R gene, which is crucial for accurate diagnosis and appropriate family screening. In this patient, the genetic mutation was identified using the next-generation sequencing (NGS) method, a relatively new technique being applied globally. This method has several advantages over the traditional Sanger method, particularly in its ability to detect novel, potentially pathogenic variants. These variants can then be added to the shared genome database [4]. The management of FH involves aggressive lipid-lowering therapy, typically with high-dose statins, ezetimibe, and PCSK9 inhibitors, to achieve target LDL-C levels. In cases of acute coronary syndrome, timely reperfusion therapy with PCI is essential to restore coronary flow and improve outcomes [16].

Recognizing the lesion as a complex LMCA true-bifurcation according to the DEFINITION II criteria (meeting one major and two minor criteria) [17], we decided to adopt a two-stent strategy from the outset. Given the patient’s cardiogenic shock and unstable hemodynamics, combined with the complexity and substantial risk of PCI, providing hemodynamic support via a mechanical circulatory device before intervention is essential. In this case, an intra-aortic balloon pump (IABP) was chosen to stabilize the patient's condition during the procedure [18]. The choice to employ the reverse TAP stenting technique was influenced by the complex nature of the LMCA true bifurcation lesion, coupled with the substantial risk of losing the LAD if stenting from the LMCA to the LCx was attempted first. Additionally, the technique’s straightforward approach and quick execution made it an ideal option in scenarios of cardiogenic shock, where avoiding prolonged procedures is crucial [19]. By employing the reverse TAP technique, we aimed to address the patient’s complex coronary anatomy effectively while minimizing procedural risks. Initially, placing the stent from the LAD to the LMCA was crucial for maintaining patency and preventing the compromise of a vital artery. Subsequently, deploying the second stent from the LMCA to the LCx, followed by final kissing balloon inflation, ensures complete coverage of the bifurcation lesion and optimal stent expansion, which reduces the risk of restenosis. This approach highlights the importance of combining advanced interventional techniques with supportive measures to manage critically ill patients with complex coronary lesions effectively. The successful outcome in this case demonstrates the efficacy of the reverse TAP technique in addressing challenging coronary bifurcation lesions and underscores the critical role of mechanical circulatory support in managing hemodynamically unstable patients during high-risk PCI procedures [12].

Familial hypercholesterolemia significantly increases the risk of recurrent cardiovascular events and is often underdiagnosed, particularly in developing regions with limited medical resources. Early recognition and intervention are critical for managing this condition effectively and preventing further complications, especially for the first relatives of index cases. In particular, screening for this genetic disorder should be indicated according to current guidelines when clinical signs are suspicious, such as AMI in young individuals, abnormally elevated serum LDL-C levels, or signs of prolonged cholesterol accumulation [20].

This case highlights the importance of clinicians being aware of FH, especially in young patients with early cardiovascular events in developing countries such as Vietnam. Detecting and treating FH promptly can lower the associated morbidity and mortality. Additionally, genetic testing should be considered for patients suspected of having FH to confirm the diagnosis and enable screening for family members.

## Conclusions

In patients with premature AMI, FH should be considered. Diagnosing FH should involve clinical tools and genetic mutation testing when available. Genetic testing not only confirms the diagnosis of FH but also facilitates pedigree screening for the patient’s relatives. For patients with complex coronary artery lesions, particularly involving the left main, the TAP technique is an appropriate choice. Larger, well-designed studies are needed to determine the prevalence of FH based on genetic mutations in young patients with acute myocardial infarction, especially in developing countries like Vietnam.
